# Raman and fluorescence micro-spectroscopy applied for the monitoring of sunitinib-loaded porous silicon nanocontainers in cardiac cells

**DOI:** 10.3389/fphar.2022.962763

**Published:** 2022-08-09

**Authors:** E. Tolstik, M. B. Gongalsky, J. Dierks, T. Brand, M. Pernecker, N. V. Pervushin, D. E. Maksutova, K. A. Gonchar, J. V. Samsonova, G. Kopeina, V. Sivakov, L. A. Osminkina, K. Lorenz

**Affiliations:** ^1^ Leibniz-Institut für Analytische Wissenschaften—ISAS—e.V., Dortmund, Germany; ^2^ Lomonosov Moscow State University, Faculty of Physics, Moscow, Russia; ^3^ Institute of Pharmacology and Toxicology, University of Würzburg, Würzburg, Germany; ^4^ Lomonosov Moscow State University, Faculty of Medicine, Moscow, Russia; ^5^ Lomonosov Moscow State University, Faculty of Chemistry, Moscow, Russia; ^6^ Leibniz Institute of Photonic Technology, Department Functional Interfaces, Jena, Germany; ^7^ Institute for Biological Instrumentation of Russian Academy of Sciences, Moscow, Russia; ^8^ Comprehensive Heart Failure Center, University Hospital of Würzburg, Würzburg, Germany

**Keywords:** porous silicon nanoparticles, sunitinib, Raman imaging, micro-spectroscopy imaging, high-resolution fluorescence microscopy, cardiomyoblast, colon cancer cells

## Abstract

Nanomaterials are a central pillar in modern medicine. They are thought to optimize drug delivery, enhance therapeutic efficacy, and reduce side-effects. To foster this technology, analytical methods are needed to validate not only the localization and distribution of these nanomaterials, but also their compatibility with cells, drugs, and drug release. In the present work, we assessed nanoparticles based on porous silicon (pSiNPs) loaded with the clinically used tyrosine kinase inhibitor sunitinib for their effectiveness of drug delivery, release, and toxicity in colon cancer cells (HCT 116 cells) and cardiac myoblast cells (H9c2) using Raman micro-spectroscopy, high-resolution fluorescence microscopy, along with biological methods for toxicological effects. We produced pSiNPs with a size of about 100 nm by grinding mesoporous silicon layers. pSiNPs allowed an effective loading of sunitinib due to their high porosity. Photoluminescence properties of the nanoparticles within the visible spectrum allowed the visualization of their uptake in cardiac cells. Raman micro-spectroscopy allowed not only the detection of the uptake and distribution of pSiNPs within the cells via a characteristic silicon Raman band at about 518–520 cm^−1^, but also the localization of the drug based on its characteristic molecular fingerprints. Cytotoxicity studies by Western blot analyses of apoptotic marker proteins such as caspase-3, and the detection of apoptosis by subG1-positive cell fractions in HCT 116 and MTT analyses in H9c2 cells, suggest a sustained release of sunitinib from pSiNPs and delayed cytotoxicity of sunitinib in HCT 116 cells. The analyses in cardiac cells revealed that pSiNPs are well tolerated and that they may even protect from toxic effects in these cells to some extent. Analyses of the integrity of mitochondrial networks as an early indicator for apoptotic cellular effects seem to validate these observations. Our study suggests pSiNPs-based nanocontainers for efficient and safe drug delivery and Raman micro-spectroscopy as a reliable method for their detection and monitoring. Thus, the herein presented nanocontainers and analytical methods have the potential to allow an efficient advancement of nanoparticles for targeted and sustained intracellular drug release that is of need, e.g., in chronic diseases and for the prevention of cardiac toxicity.

## 1 Introduction

The application of novel bioimaging techniques and high-resolution optical modalities supports the understanding of cellular distribution and the drug’s effects on organelles and cellular dynamics. This is particularly relevant for cardiomyocytes as these are non-dividing cells that are especially vulnerable. Depending on the respective drug, cardiotoxicity impacts, e.g., on mitochondrial function, cardiomyocyte survival, oxidative stress levels, DNA damage, endothelial cell permeability, or disturbance of the conduction system, leading to reversible or irreversible damages. Overall, cardiotoxicity is one of the most significant adverse effects of drugs in general and of chemotherapeutics in particular and strongly impacts on morbidity and mortality of the cancer patients (Pai and Nahata, 2000; Palmer et al., 2020). Hence, there is an urgent need to understand the cell-type-specific localization of antitumor drugs on heart cells and to use the knowledge for the reduction or circumvention of cardiotoxic side-effects of anticancer therapies.

The search for new and innovative ways to reduce cardiac side-effects via optimized drug uptake and release is one of the major challenges in the field of cardio-oncology (Chu et al., 2007). To prevent chemotherapy from affecting the whole body, novel drug delivery systems based on nanocontainers for the transport for the sustained and/or the targeted release of the drug have been developed (Yao et al., 2020). Especially, sustained drug release, i.e. delayed release, allows a particular drug to be delivered at a programmed rate, leading therewith to a longer and controlled drug impact on the body avoiding toxic peak concentrations of a drug (Moghimi et al., 2001). Drug delivery based on nanoparticles has several advantages over the delivery of free chemotherapeutic agents (Fornaguera and García-Celma, 2017): the small size of the containers (less than 200 nm) enables efficient intracellular drug delivery and controlled release, a large surface-to-volume ratio increases drug payload in case of porous nanocontainers, and a specific surface activation allows targeted drug delivery. In addition to therapy, the same nanoparticles can also be used for diagnostics to monitor the distribution of nanoparticles as contrast agents (Park et al., 2009). Among the available nanomaterials, porous silicon nanoparticles (pSiNPs) belong to promising delivery vehicles due to their high drug-loading capacity, biocompatibility, and biodegradability (Fornaguera and García-Celma, 2017; Wan et al., 2018). The pSiNPs are degraded to non-toxic and well-tolerated silicic acid species (Anderson et al., 2003; Park et al., 2009; Gu et al., 2013; Gongalsky et al., 2020). Moreover, the morphology and porosity of pSiNPs can easily be adapted by modification of certain fabrication parameters (Herino et al., 1987; Lehmann et al., 2000). The intrinsic photoluminescence (PL) properties of pSiNPs, which consist of silicon quantum dots and pores, provide sufficient contrast for fluorescent bio-visualization both *in vitro* and *in vivo* (Osminkina et al., 2012; Peng et al., 2013).

Recently, vibrational spectroscopy like Raman micro-spectroscopy (Pliss et al., 2010; Bocklitz et al., 2016; Petersen et al., 2017; Yosef et al., 2017; Guo et al., 2018; Kirchberger-Tolstik et al., 2020; Gongalsky et al., 2021a; Tolstik et al., 2022) has emerged as a powerful and well-suited tool in bioanalytics. Raman spectroscopy detects molecular vibrations, which are activated by laser radiation. Based on Raman spectra, chemical fingerprints of the molecules can be determined (Krafft et al., 2009), which allow the localization of substances within a cell. Raman micro-spectroscopy allows spectroscopic monitoring of the uptake, intracellular localization, and dissolution of pSiNPs in living tumor cells through the detection of a specific Raman band at 520 cm^−1^ based on crystalline silicon band (Tolstik et al., 2016b; Tolstik et al., 2016a). Biodegradation of pSiNPs is accompanied by a decrease in the size of silicon nanocrystals and their oxidation and, according to the quantum соnfinement effect, is detected as a low-frequency shift with a significant decrease in the intensity and broadening of the Raman signal (Tolstik et al., 2016b; Gongalsky et al., 2021a). Raman spectroscopy also enables time-resolved 3D mapping of the distribution of drugs and nanoparticle drug reservoirs (Chernenko et al., 2009; Gordon and McGoverin, 2011; Matthäus et al., 2011; Choi et al., 2013). Differences in the chemical composition of certain cellular compartments allow to localize drugs at subcellular resolution and thus achieve a sophisticated evaluation of drug uptake and its subsequent release (Matthaus et al., 2008; Krafft et al., 2009; Peng et al., 2014; Eberhardt et al., 2015; Tolstik et al., 2016b).

In the present study, we focused on an orally active compound sunitinib malate (SU), a multi-targeted tyrosine kinase inhibitor, approved by the FDA as therapy for renal cell carcinoma and gastrointestinal stromal tumors (Chu et al., 2007). It has also been used for other malignancies, including colon cancer, breast cancer, neuroendocrine cancer, and lung cancer in preclinical and clinical trials (Demetri et al., 2006; Motzer et al., 2007). Along with the fact that cancer patients undergoing successful chemotherapy with SU often have an improved overall survival, a wide range of severe cardiac side-effects have been reported that lead to severe symptomatic heart failure in about 2% of the patients (Gorini et al., 2018; Silva et al., 2018; Palmer et al., 2020). SU has a poor solubility in water, which leads to the instability of its therapeutic effects (Alshehri and Shakeel, 2020). To increase the solubility and bioavailability of SU, it was proposed to deliver it in nanoform (Chakravarty et al., 2015). Thereby, it is crucial to study the interaction of SU with the respective nanocontainers to investigate the benefits of SU delivery using a nanoform compared to pure drug delivery, along with its impact on cells *in vitro*.

In the current work, we assessed the use of pSiNPs loaded with SU to evaluate the effectiveness of drug delivery, release, and its toxicity on cancer and cardiac cells using several imaging modalities including confocal fluorescence microscopy with high spatial resolution and Raman micro-spectroscopy, along with the biological methods for toxicological and morphological studies. The choice of pSiNPs as nanocontainers is driven by their proven unique physico-chemical properties: biocompatibility, biodegradability, and high loading efficiency for drug delivery. Moreover, PL properties of pSiNPs enable fast intracellular imaging and mapping of the SU loaded in nanocontainers applying standard fluorescence staining techniques. The presence of characteristic Raman modes of SU (Litti et al., 2016) and pSiNPs (Tolstik et al., 2016b) can facilitate its label-free tracking by applying Raman spectroscopy. For *in vitro* studies, to evaluate the cardiotoxicity of SU-loaded pSiNPs, the following two cell lines were chosen: the human colon carcinoma HCT 116 cell line (Rajput et al., 2008) that is applied widely as a model to study the cellular effects of SU (Sun et al., 2012; Ban et al., 2017; Elgendy et al., 2017) and the rat cardiomyoblast cell line H9c2 (Korashy et al., 2015; Tomasovic et al., 2020; Merches et al., 2022). Thereby, the intracellular delivery, localization, and toxicity of pure SU substance and SU-loaded pSiNPs were analyzed *in vitro* using cell viability assays and linear optical imaging techniques.

## 2 Materials and methods

### 2.1 Porous silicon nanoparticles preparation

Porous silicon (pSi) layers were prepared by anodizing p-type heavily boron-doped (100)-oriented silicon (SiMat, Germany) wafers with resistivity of 0.001–0.002 Ω cm in a hydrogen fluoride (HF, CAS 7664-39-3, 48%, Merck)/ethanol (CAS 64-17-5, ≥99.8%, VWR Chemicals) mixture 1:1 v/v and current density of 50 mA/cm^2^ for 1 h. The obtained porous layers were separated from the wafer by applying 3 pulses of current density of 600 mA/cm^2^ for 3 s. After drying at room temperature (RT) overnight, the layers were ground in agar mortar into millimeter-sized particles. The obtained particles were then ball milled in deionized water in the planetary mill (Fritsch Pulverisette 7 premium line, FRITSCH GmbH) for 30 min with zirconium oxide balls of diameter 2.5 μm and then for 30 min with balls of diameter 0.1 μm. Nanoparticles (NPs) were collected from the sample by centrifugation at 12,500 rpm for 20 min, leaving the supernatant for further work. The obtained pSiNPs were stored in deionized water.

### 2.2 Nanoparticle characterization

Structural analysis of the pSiNPs was carried out by transmission electron microscopy (TEM, LEO912 AB OMEGA). The samples of pSiNPs for TEM studies were prepared by deposition of a drop of the aqueous suspension of NPs on standard carbon-coated copper TEM-grids followed by air drying for 10 min. The dynamic light scattering (DLS) measurements were performed with a Malvern Zetasizer Nano ZS instrument to determine the size distribution and zeta potential (ZP) of NPs in aqueous suspensions. Nanoparticles with a zeta potential between −10 and +10 mV were considered approximately neutral, while nanoparticles with zeta potential of greater than +30 mV or less than −30 mV were considered strongly cationic and strongly anionic, respectively. In addition, zeta potential of the surface reflects the solubility and stability of colloidal NPs. The infrared transmission spectra of pSiNPs were measured on a Bruker IFS 66 v/S (FTIR) infrared spectrometer with Fourier transformation, for which a drop of suspension was dried on a silicon high-resistance substrate with two-sided polishing. Raman micro-spectroscopy data were acquired using a confocal Raman microscope Confotec™ MR350 system, with a laser excitation at 633 nm wavelength and 0.5 mW power.

### 2.3 Cell culture and experimental procedures

Cell culture experiments were performed using human colorectal carcinoma HCT 116 cell line, which was provided by the Department of Toxicology of the Karolinska Institute (Stockholm, Sweden), and rat cardiac myoblast H9c2 cells (ATTC, Manassas, VA, United States). The HCT 116 cells were grown in 5% CO_2_ at 37°C, in Dulbecco’s Modified Eagle Medium (DMEM) with high glucose (Gibco, Waltham, MA, United States), supplemented by 10% fetal bovine serum (Gibco), 1 mM sodium pyruvate (PanEco, Moscow, Russia), and a mixture of antibiotics and antimycotics (penicillin, streptomycin, and amphotericin B; CAS 15240-062, Gibco). The rat cardiomyoblast cell line H9c2 (CRL-1446™, ATCC, United States) was cultured at 37°C under a humidified atmosphere of 5% CO_2_ in DMEM (P04-03596, Pan Biotech, Germany) supplemented with 10% fetal calf serum (FCS; S0115, Sigma Aldrich, United Kingdom), 100 U ml^−1^ penicillin G and 100 μg ml^−1^ streptomycin sulfate (P06-07100, Pan Biotech, Germany), and 2 mM l-glutamine (P04-80100, Pan Biotech, Germany). All cells were reseeded every 3 days and maintained in the logarithmic growth phase for experiments. Before treatment with nanoparticles and/or SU malate (Sigma-Aldrich, St. Louis, MO, United States, >98% (HPLC)), the culture medium was replaced with fresh medium. Cells were treated with nanoparticles at a concentration of 200 μg/mL, SU malate at 5–50 μM, and nanoparticles loaded with SU malate at the same concentrations. Then, the cells were incubated for 24–120 h with various concentrations of SU.

### 2.4 Drug loading and release

An aqueous solution of 0.2 mM sunitinib malate (SU, ≥98% (HPLC), Sigma-Aldrich, Germany) was mixed with 1 mg/ml pSiNPs overnight. Unbound SU was removed by two centrifugation steps: removal of the supernatant and resuspension in water. The SU loading percentage was calculated according to the formula *100%·(I*_*ini*_ *–I*_*s1*_ *–I*_*s2*_ *)/I*_*ini*_, where *I*_*ini*_ is the absorption intensity of the initial solution and *I*_*s1*_ and *I*_*s2*_ are the absorption intensities of the supernatants after the first and second centrifugations, respectively. SU release from pSiNPs was analyzed in PBS at 37°C. At pre-defined time intervals, the samples were centrifuged and the supernatant with the released SU was taken in Varioscan flash (Thermo Fisher Scientific, United States) at 429 nm. The spectrophotometer Genesys 20 (4001/4, Thermo Fisher Scientific Inc., Oberhausen, Germany) was used to determine the concentration of SU in a sample by measuring the absorption of visible light in the sample.

### 2.5 Gel electrophoresis and Western blot analysis

HCT 116 and H9c2 cells were lysed using lysis buffer containing 1% (v/v) Triton-X-100, 5 mM EDTA, 300 mM NaCl, 50 mM Tris (pH 7.4), 20 μg/ml soybean trypsin inhibitor, 0.4 mM benzamidine, 1 mM PMSF, 50 mM NaF, 5 mM Na_4_P_3_O_7_, 1 mM Na_3_VO_4_, and 1.5 mM NaN_3_ on ice for 20 min. Next, cells were centrifuged (1000 rcf, 4 min, +4°C) and washed twice with ice-cold phosphate-buffered saline (PBS) solution (PanEco). Then, the pellet was lysed in radioimmunoprecipitation assay (RIPA) buffer, which contains 50 mM Tris-HCl (pH 7.4), 150 mM NaCl, 2 mM EDTA, 0.5% SDS, 0.5% sodium deoxycholate, 1% NP-40, 1 mM phenylmethylsulfonyl fluoride (PMSF), and cOmplete™ Protease Inhibitor Cocktail (Roche, Basel, Switzerland), for 20 min on ice. After centrifugation (15,000 rcf, 15 min, +4°C), a part of the supernatant was taken for protein concentration assay, and another part was used for Western blot (WB) analysis, as previously described (Gongalsky et al., 2021b).

The following primary antibodies were used for WB: anti-rabbit full and cleaved caspase-3 (#9662) antibodies (Cell Signaling Technology, Danvers, MA, United States); Gβ (1:5,000; sc-378; Santa Cruz Biotechnology); and anti-poly(ADP ribose) polymerase (PARP) (#137653 or ab137653) and anti-mouse tubulin-a (#7291) (both from Abcam, Cambridge, United Kingdom). Horseradish peroxidase (HRP)-linked goat anti-mouse and anti-rabbit antibodies (#97046 and #97200, respectively; Abcam) were used as secondary antibodies. For detection, Pierce ECL Plus Kit was used (32132, Thermo Fisher Scientific Inc., United States).

### 2.6 SubG1 test of HCT 116 cells

After the indicated time of treatment, cells were collected and fixed in 70% ethanol during 1 h at −20°C. Next, ethanol was washed off and the cells were re-suspended in PBS, supplemented with 1% RNase A, and stained with 20 μg/ml propidium iodide (PI) for 15 min at 37°C. After staining, the cells were examined using the FACSCanto II cell analyzer (BD Biosciences).

### 2.7 MTT assay of H9c2 cells

The MTT (3-(4,5-dimethylthiazol-2-yl)-2,5-diphenyl-tetrazolium bromide) assay assesses the cell metabolic activity as an indicator for cell viability. For the assay, the cell culture medium was removed and replaced with 0.5 mg ml^−1^ MTT (M5655, Sigma-Aldrich, United States) dissolved in the respective cell culture medium. After 2 h at 37°C and 5% CO_2_, the staining solution was discarded and the cells were lysed using 0.1 M HCl diluted in isopropanol (CP41.2, Carl Roth, Germany). For quantification, the cell lysate was transferred to a 96-well plate and the absorbance measured at 570 nm with reference at 650 nm using a microplate reader (Spark 20M, Tecan, Switzerland). The measured values were normalized to untreated control cells.

### 2.8 Cell staining for fluorescence microscopy

For fluorescence imaging, the H9c2 and HCT 116 cells were grown on the coverslips in standard 10 cm Petri dishes filled with DMEM culture medium. When cells reached the desired confluency, the medium was replaced with fresh medium. The cells were treated with nanoparticles at a concentration of 200 μg/ml, SU malate at 5–50 μM, and nanoparticles loaded with SU malate at the same concentrations. The cells were treated for indicated time intervals. The medium was removed from the dishes, and pre-warmed (37°C) staining solutions containing MitoTracker^®^ Orange (100 nM) (Thermo Fischer Scientific Inc., United States) were added for 1 h under growth conditions (at 37°C and 5% CO_2_); all cells were washed two times for 3 min with PBS and fixed in 4% paraformaldehyde (PFA) solution for 10 min. If required, an additional staining of the cytoskeleton was performed: the cells were incubated in 0.2 vol.% Triton X-100 for 10 min to permeabilize the cell membrane and thereafter stained with Alexa Fluor 488 Phalloidin (Thermo Fischer Scientific Inc., United States) for 45 min. At the end, the stained cells were fixed on the microscope slides (Epredia™ SuperFrost Plus™ Adhesion slides 75 × 25 mm, Lauda Königshofen, Germany) by Fluoromount-G^®^ and dried overnight.

### 2.9 Confocal fluorescence imaging and mitochondrial analyses

Fluorescence microscopy images were acquired using a fluorescence microscope (TCS SP8 DLS, Leica Microsystems, Germany). Lasers with several excitation wavelengths were applied simultaneously for various staining of the samples: a 488-nm laser was used to activate Alexa Fluor 488 Phalloidin, a 552-nm laser for MitoTracker^®^ Orange CMTMRos, and a 638-nm laser for the pSiNPs. As was mentioned earlier, PL properties of pSiNPs in the range of 600–800 nm allow a label-free activation and determination of the nanocontainers (Tolstik et al., 2016a). The LAS X software (Leica Application Suite X, version: 3.5.7.23225) was utilized for the data acquisition. All cells were measured using a 63×/1.3 NA oil objective (Zeiss, Germany). The images format was 1024 × 1024 pixels and the speed of image acquisition was 100 Hz. For the quantitative analysis of the mitochondrial structures, Fiji program was used to preprocess the measurements, to create a segmentation map of the mitochondria, and to perform a binary skeleton analysis, following the protocol presented by Haupt et al. (2022) (Schindelin et al., 2012). Based on this, the percentage of branched mitochondrial networks was calculated and compared through the different concentrations.

### 2.10 Raman micro-spectroscopy imaging, data preprocessing, and data analyses

Raman micro-spectroscopy imaging of H9c2 cells was performed using a confocal Raman microscope (alpha300R, WITec, Germany). The software WITec Control FIVE (version: 5.3.12.104), a laser source at 785-nm excitation wavelength, 200 mW power, 7–8 s integration time, 300 g/mm based spectrometer grating, and 63×/1.0 NA water immersion objective (Zeiss, Germany) were utilized for data acquisition. Spatial resolution was 0.5 μm/pixel for all three dimensions. Additionally, crystallized SU (Cayman Chemical, Michigan, United States) was measured as a reference spectrum with 100 mW power, 30 s integration time, 20 accumulations, and a 50×/0.75 NA dry objective (Zeiss, Germany). Additionally, Raman spectra of the solutions of pure pSiNPs and pSiNPs loaded with SU (dissolved in deionized H_2_O) were measured on CaF_2_ slides. The WITec software (Control FIVE 5.3.12.104) was used for data acquisition.

Data preprocessing and reconstruction of all spectral measurements were implemented and performed in Python 3 (version 3.9.5) programming language, using mainly “numpy,” “scipy,” and “scikit-learn” for computation and “matplotlib” for visualization tools. The data were preprocessed by homemade algorithms, including a cosmic spike correction (Ryabchykov et al., 2016), baseline correction using sensitive nonlinear iterative peak (SNIP) clipping (Morháč and Matoušek, 2008), Savitzky–Golay filtering, background segmentation in case of cell images, spectral truncation to Raman fingerprint region, and vector normalization. After data preprocessing, a combination of principal component analysis (PCA) and hierarchical cluster analysis (HCA) was applied, aiming to identify the drug intracellularly, combining cell spectra in representable groups (Pearson, 1901; Hierarchical Cluster Analysis - Cecil C. Bridges, 1966; Wold et al., 1987). The clustering was performed on all selected data at once, where a mix of untreated cells was compared to cells treated with pure SU or cells treated with SU loaded in pSiNPs. The resulting mean spectra were evaluated manually and compared to the measured reference spectra. Also, the number of clusters for HCA was set manually as low as possible to still detect a meaningful difference between the groups. In addition, the spectra were divided with respect to different spectral meaning and not by different measurement properties and conditions. Based on this, a reasonable amount of clusters (e.g., nucleus, cytoplasm, and, in rare cases, clusters with mitochondrial contribution) was expected to be found in both groups.

## 3 Result and discussion

### 3.1 pSiNPs characterization

**FIGURE 1:**
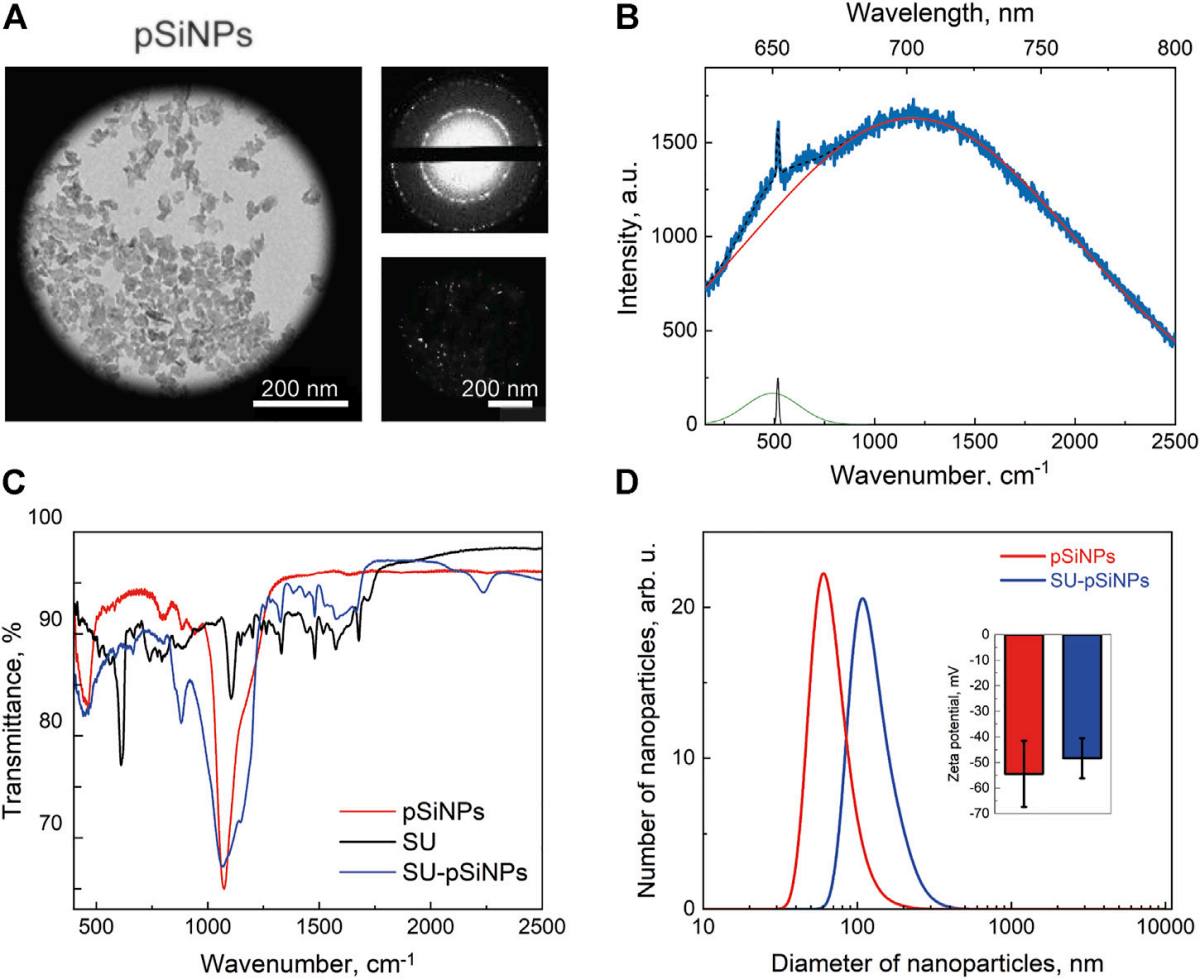
Characterization of pSiNPs. **(A)** TEM image of pSiNPs (left) with an electron diffraction pattern (top small picture) and a dark-field image (bottom small picture). **(B)** PL and Raman spectra of pSiNPs. Experimental spectrum (blue line) and its deconvolution, i.e., resulted spectrum (dashed black line) and components: PL (red line), Raman amorphous Si (480 cm^−1^—green line), and nc-Si (518 cm^−1^—black line) peaks. Laser excitation wavelength is 633 nm. **(C)** FTIR spectrum of pSiNPs, SU, and SU-pSiNPs. **(D)** Diameter distribution of pSiNPs before (red) and after (blue) loading of SU in aqueous suspension measured by means of dynamic light scattering. The inset shows zeta potentials of pSiNPs.

For the assessment of the compatibility of pSiNPs in cancer and cardiac cells, pSiNPs are required to fulfill certain quality measures, such as nanoparticle diameter of less than 200 nm for effective internalization into the cells, a porous structure for an effective drug loading and drug delivery, and the presence of PL properties and nanocrystallinity to be well identified by luminescence and Raman microscopy methods (Osminkina and Gongalsky, 2018). To achieve these characteristics, pSiNPs were prepared by electrochemical etching of crystalline silicon substrate in HF and ethanol solution, lifting off the obtained porous silicon layer, ball-milling, and centrifugation. The preparation conditions were optimized to provide pore size and surface area of the nanoparticles suitable for efficient loading of chemotherapeutics while maintaining an acceptable pSiNPs biodegradation rate (Maximchik et al., 2019). According to TEM micrographs presented in Figure 1A, nanoparticles of about 100 nm in diameter were achieved, with an irregular shape due to the top-down fabrication process and consisting of smaller silicon nanocrystals (nc-Si) and pores. The crystallinity of pSiNPs was confirmed by the pattern of nc-Si electron diffraction, which contains both diffraction rings and bright spots, along with dark-field TEM images, where bright spots from nc-Si were also observed (Figure 1A, *top and bottom images*) (Osminkina and Gongalsky, 2018).

The PL spectrum of the pSiNPs is shown together with their Raman spectrum in Figure 1B. The experimentally obtained spectrum (blue line) was approximated by superposition of the wide Gaussian PL band (red line) and Raman spectrum (black line). Deconvolution of the Raman spectrum showed two maxima corresponding to amorphous Si at 480 cm^−1^ and nc-Si at 518 cm^−1^ (Tolstik et al., 2016b). The RT PL band has a maximum at 690 nm that is well explained by the radiative recombination of excitons confined in nc-Si with an average size of about 3.5 nm. The exact position of the Raman band was determined by the average diameter of the Si nanocrystals, which represent the size of about 3.6 nm (Gongalsky et al., 2020; 2021b).

Figure 1C shows FTIR spectra for pSiNPs, SU, and SU-loaded pSiNPs (SU-pSiNPs). The surface of pSiNPs is oxidized in either the air atmosphere or the aqueous suspension. That is, nc-Si core is surrounded by SiO_x_ shell with possible amorphous Si interface between them. The oxidation of pSiNPs is visible in Figure 1C by IR absorption bands, which correspond to the δSi–O–Si deformation vibration mode at 484 cm^−1^, to Si_x_O_y_ vibration mode at 800 cm^−1^, and to νSi–O–Si vibration mode of the transversal optic modes at 1060 cm^−1^ and longitudinal optic phonons at 1200 cm^−1^. This oxygen coating of nanoparticles provides a negative charge on their surface and their hydrophilic properties (Sailor, 2022). The FTIR spectrum of SU contains a large number of components responsible for the absorption of IR radiation by various vibrations of bonds within the molecule (Tarasi et al., 2022). It should be noted that the spectrum of SU-pSiNPs contains both the absorption bands of silicon–oxygen bonds described earlier and the absorption bands of SU. This testifies to the effective adsorption of SU in the pores of pSiNPs.

**FIGURE 2:**
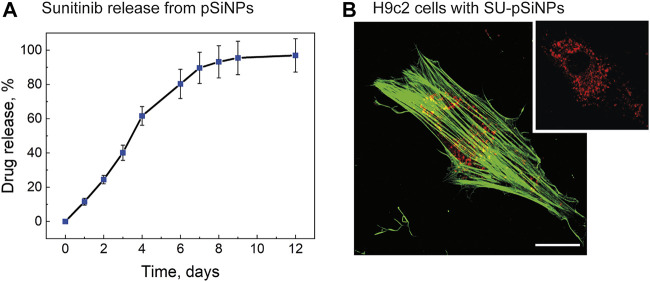
**(A)** Drug release profile of SU loaded into pSiNPs over time (days). Typical release time (50% of payload) is demonstrated after 4 days. **(B)** A representative fluorescence image of H9c2 cell treated with SU-loaded nanoparticles for 24 h; the content of SU in the particles was 20 μM. The cytoskeleton was labeled with Alexa Fluor 488 Phalloidin and is depicted in green. For the visualization of pSiNPs, the laser at 638-nm excitation wavelength was used; the pSiNPs are depicted in red. SU could not be detected with the fluorescence microscopy without labeling. The scale bar represents 20 μm.

Next, we monitored the drug release from SU-loaded pSiNPs (Figure 2A). The typical release duration (50% of payload) was 4 days, which can be considered as sustained release. SU retention in pSiNPs is most likely due to the “electrostatic adsorption” or coulombic forces between negative SiO_2_ surface of pSiNPs and the positively charged SU molecules (Anglin et al., 2008), and the moderate solubility of SU malate in aqueous medium (Alshehri and Shakeel, 2020).

For the loading of the pSiNPs with SU, SU was stirred together with the pSiNPs in water for 24 h at RT. After loading, the unbound drug was removed by a washing process using two centrifugation steps. The SU loading efficiency into the pSiNPs was determined by comparing the optical transmission intensities of the initial drug solution and the supernatant after loading and was about 45% of the initial concentration of the drug solution. The mean hydrodynamic size of pSiNPs, measured by DLS, was consistent with the TEM measurements (Figure 1D). SU loading leads to an increase in the size of pSiNPs (about 185 nm). This may be explained by the positive charge of SU, which can partially neutralize the negative charge of the pSiNPs and thereby increase their van der Waals interactions. It is clearly visible in Figure 1D; the presented statistically non-significant change in the value of zeta potential shows that the particles partially lose their negative charge when they are loaded with SU.

To test the uptake and distribution of SU-pSiNPs intracellularly, the cardiac myoblast cell line H9c2 was investigated as the heart has been reported to be a target of the drug’s side-effect by SU therapy. The cells were incubated with SU-pSiNPs for 24 h. To monitor the SU-pSiNPs localization, the actin filaments of the cytoskeleton were stained with Alexa Fluor 488 Phalloidin. Due to their strong PL features, pSiNPs possess well-visible fluorescence properties, with absorption in the range of 600–800 nm (see Figure 1B). A representative confocal fluorescence image in Figure 2B demonstrates that pSiNPs (depicted in red) effectively penetrated into the cells within 1 day of incubation and localized mostly inside the cytoplasm. Thus, the uptake of the nanocontainers seems efficient and can easily be detected by fluorescence microscopy. However, no detection of the drug and its release could be studied by standard confocal fluorescence imaging. Commonly used methods, e.g., immunofluorescence or mass spectrometry, do not also allow a longitudinal monitoring of the drug release. Thus, confocal Raman micro-spectroscopy can have decisive advantages here, as the drug distribution in the cells can be traced without labeling (Aljakouch et al., 2018; El-Mashtoly, 2020). For example, the release of doxorubicin from pSiNPs in cancer cells was precisely visualized in our previous studies by applying Raman spectroscopy (Maximchik et al., 2019).

### 3.2 Raman micro-spectroscopy studies of sunitinib, pSiNPs, and SU-pSiNPs *in vitro*

**FIGURE 3:**
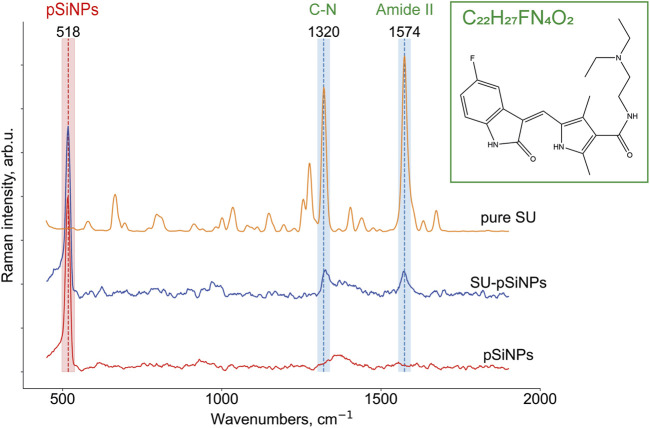
Raman reference spectra of crystallized SU (C_22_H_27_FN_4_O_2_; *N*-[2-(diethylamino)ethyl]-5-[(*Z*)-(5-fluor-1,2-dihydro-2-oxo-3H-indol-3-ylidene)-methyl]-2,4-dimethyl-1H-pyrrole-3-carboxamide), depicted in orange, of solutions of SU loaded in pSiNPs (SU-pSiNPs, depicted in blue) and of pure Si nanocontainers (pSiNPs, depicted in red) dissolved in deionized water for 24 h. The Raman spectra were preprocessed (see Section 2 for the details) and the characteristic Raman bands at 518 cm^−1^ for pSiNPs, and 1320 cm^−1^ and 1574 cm^−1^ for SU, respectively, were specified.

To localize pSiNPs, SU, and SU-pSiNPs intracellularly, Raman imaging was utilized. Raman micro-spectroscopy allows the detection of compounds based on their biochemical differences within biological samples. The advantage of this label-free method is in the possibility to identify molecules and compounds based on their molecular fingerprints, i.e., molecular vibrations. To our knowledge, there are no studies performed on the interaction of pSiNPs and cardiac cells. Using Raman spectroscopy can prove the uptake of pSiNPs by cardiomyocytes and the safety of these nanocontainers as “transporters” of cargo into cardiomyoblasts. Thus, porous silicon nanoparticles promise to be a safe and effective delivery system for drugs like SU in cardiomyoblasts. Thereby, aiming to detect the localization of the drug and nano-containers intracellularly, we first recorded Raman reference spectra of crystalline SU, pSiNPs, and SU-pSiNPs (Figure 3)*.* SU (depicted in orange) contains dominant intensity peaks at 1320 cm^−1^ and 1574 cm^−1^ that are associated with C–N and amide stretching vibrations, respectively (Litti et al., 2016). It is known that the amide II band consists of 60% N–H and 40% C–N stretches and is presented near 1550 cm^−1^ and the amide III band consists of 40% C–N and 30% N–H stretches and is presented near 1300 cm^−1^ (Horiba, 2021). The predominant Raman band of pSiNPs (depicted in red) was slightly shifted from the characteristic wavenumber at 520 cm^−1^ to 518 cm^−1^. This shift is explained by the dissolution processes of pSiNPs, as this is associated with a decrease in the Si crystal size and therewith a Raman band shift to the lower wavenumbers (Tolstik et al., 2016b). The reference Raman spectrum of SU-pSiNPs in aqueous solution (depicted in blue) possesses Raman bands of both molecules: SU-associated stretching vibrations at 1324 cm^−1^ and 1572 cm^−1^ and the silicon signal at 518 cm^−1^.

**FIGURE 4:**
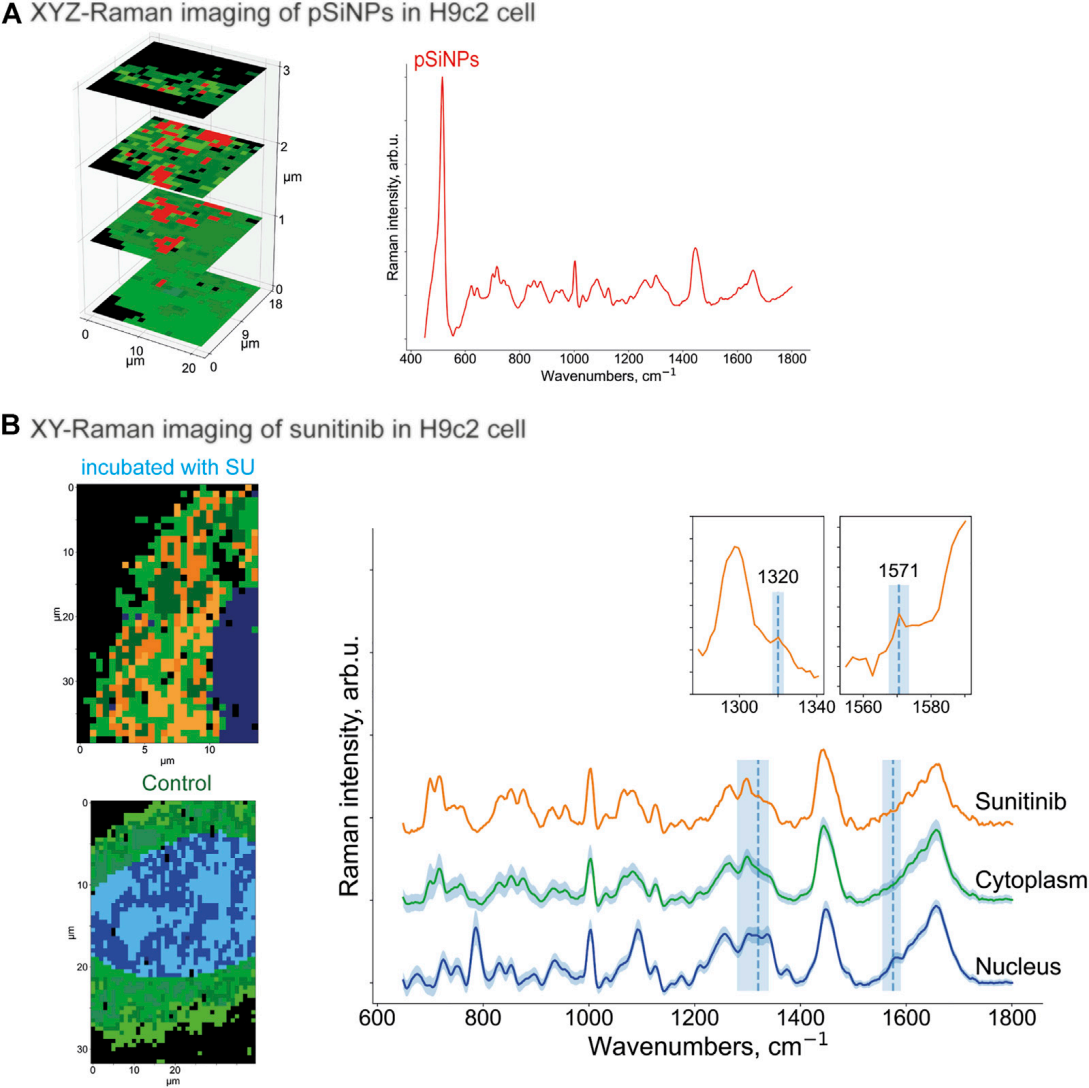
**(A)** XYZ-Raman micro-spectroscopy image within a H9c2 cell incubated with pSiNPs in a concentration of 200 μg/ml. The spectrum of the cluster that corresponds to the pSiNPs has a Si-specific peak at 518 cm^−1^ and is depicted in red; the cluster that corresponds to cytoplasm is depicted in green shades. **(B)** XY-Raman micro-spectroscopy images of H9c2 cell incubated with 20 μM of SU for 24 h (upper image, left), control H9c2 cell (lower image, left) and the corresponding Raman spectra of three dominant clusters with sunitinib, cytoplasmic, and nuleus inputs. Thus, after data preprocessing, HCA clustering was applied to distinguish the following main components: cytoplasm (multiple green colors), nucleus (multiple blue colors), and orange containing SU with characteristic Raman bands at 1320 cm^−1^ and 1574 cm^−1^. While the peak at 1320 cm^−1^ is only minor ones, the Raman band at 1574 cm^−1^ is more pronounced. Both SU-specific peaks have low intensity compared to the reference spectra, and the orange SU cluster also has a partial overlap with the cell interior signals (characteristic Raman bands at 1450 cm^−1^ and 1650 cm^−1^).

To prove the intracellular uptake of SU with and without nanocontainers, we incubated the H9c2 cells with pure SU and with unloaded pSiNPs for 24–72 h using concentrations of SU in the first case between 5 and 50 μM. First, Raman spectroscopy images of H9c2 cells incubated with pure pSiNPs were recorded by performing a 3D scan over several XY-layers within the cell with 1 μm step in all spatial dimensions aiming to detect intracellular accumulations of nanoparticles. The representative 3D image for the pSiNPs in a concentration of 200 μg/ml is demonstrated in Figure 4A. The dominant Raman band of nc-Si at 518 cm^−1^ enables the separation of clusters containing pSiNPs (depicted in red) from clusters containing signals of the cytoplasm (for simplicity, here all spectra of cytosolic cellular components are depicted in green shades). This 3D scan of a cytoplasmic part of the cell covering 3 μm shows that pSiNPs have indeed passed the cell membrane and have entered the cytoplasm. It suggests an efficient uptake of the nanoparticles into the cytoplasm, which confirmed the results obtained by fluorescence microscopy (Figure 2B). Moreover, as the interior part of the cell was measured in z-direction, the localization of pSiNPs was primarily referred to the cytoplasm with no Si accumulations at the cell membrane. Comparable incubation steps followed by Raman imaging were also performed using HCT 116 cells with similar results.

To localize the anticancer drug SU within the cell interior, Raman imaging of cells incubated with SU (20 μM) was performed, followed by data preprocessing and hyperspectral analyses (Figure 4B). Using the clustering algorithms described in the Methods section, the clusters corresponding to SU, cytoplasm, and nuclei were identified. Thus, SU enrichments were detected throughout the cell interior based on the SU-associated Raman bands as depicted in Figure 4B as orange cluster, which are in good agreement with the ones in the reference Raman spectra of SU (see Figure 3). Both Raman bands at 1320 cm^−1^ and 1571 cm^−1^ are clearly visible; however, the intensities are low compared to the cell interior signal (characteristic stretching vibrations at 1450 cm^−1^ represent CH_2_/CH_3_ groups bending for proteins and CH scissoring for lipids; and 1650 cm^−1^ stretching vibrations represent random coil of amide 1 for proteins and C=C stretching for lipids) (Risi et al., 2012; Tolstik et al., 2016b). The reason is the low abundance of SU compared to the cell components. It is hardly possible to unambiguously visualize pure SU molecules intracellularly with the provided resolution of the microscope and herein used concentrations. However, we succeeded in visualizing SU-associated enrichments intracellularly. The spectra of the nucleus and the cytoplasm are depicted in blue and green, respectively. The majority of the SU-assigned pixels were located within the cytoplasm and did not penetrate into the nucleus. The comparison of these measurements with those in control H9c2 cells (Figure 4B, below) validated the detection of SU-associated Raman bands only in the SU-treated cells. Taken together, we showed that Raman imaging allowed the detection of compounds, nanocontainers, or cellular components without applying additional staining procedures or chemical modifications, which may allow the longitudinal and non-invasive monitoring of the kinetics of the drugs within the cells.

**FIGURE 5:**
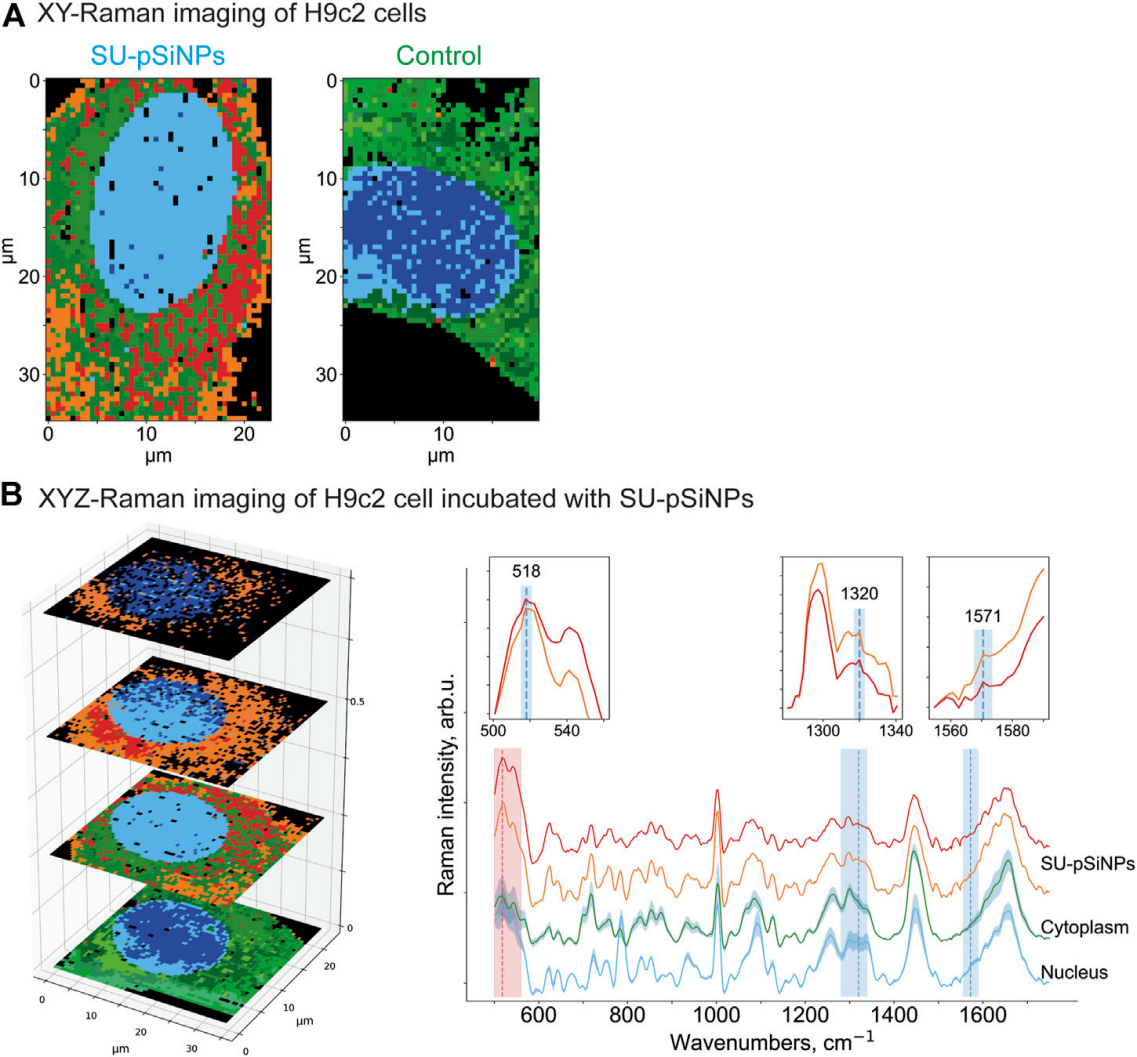
**(A)** XY- and **(B)** XYZ-Raman micro-spectroscopy images of a H9c2 cell incubated with 20 μM of SU loaded in pSiNPs for 24 h [**(A)**, left] and of a control H9c2 cell [**(A)**, right]. After data preprocessing, HCA clustering was applied to distinguish the following main components: pure cytoplasmic components (multiple green colors), nucleus (multiple blue colors), and red and orange clusters containing—next to cytoplasmic signal—SU and nanocontainer-specific peaks at 518 cm^−1^ for pSiNPs, and 1320 cm^−1^ and 1571 cm^−1^ for SU, respectively. The clusters were plotted with standard deviations shown as shadows.

Further, the H9c2 cells were incubated with SU-loaded nanocontainers. The cells treated with SU-pSiNPs and untreated cells were then compared by applying multivariate data analyses (HCA clustering; see Methods section) to separate the spectra in several clusters of interest. Thereby, SU-pSiNPs-treated cells were clustered with a ground-truth set (a set of control cells for comparison) of up to eight randomly selected untreated cells at once to find component-specific clusters in both groups. The assumption was that components that only appear in treated cells will result in unique clusters of treated cells. Also, we aimed to hold the total number of data points equal for both groups and balanced the number of control cells based on the number of data points in the treated cells. Here, the nucleus (identified by the DNA/RNA-specific band at 785 cm^−1^) was depicted in different shades of blue, while different shades of green referred to cytoplasmic clusters and are presented in 2D scans of both SU-pSiNP-treated and untreated groups (Figure 5A). For the identification of clusters as potential candidates for cellular spectra of pSiNPs or SU, the proportion of all clusters per group was determined and the clusters that are represented only in the treated group were taken for further investigation. Due to low drug concentrations and the drug’s strong spectral overlap with the Raman bands of Si and cell organelles, the differentiation of pure SU spectra with the presented optical resolution was challenging. However, two different clusters encoding SU or SU-pSiNPs were clearly separated in the treated group (Figure 5A, left panel) and are depicted in orange and red, combining cytoplasmic background and SU-pSiNPs-characteristic Raman bands. For comparison, an untreated cell was also analyzed and clustered and is shown in Figure 5B, right panel. The dominant clusters, depicted in shades of green, correspond to the cytoplasmic background; however, no characteristic Raman shifts for SU, SU-pSiNPs, or pSiNPs were detected. Also, it is visible that both orange and red clusters cannot be identified in the control group, supporting our assumption that the cluster is only present in the treated group, which thus validates our data analysis.

In addition, to confirm the localization of the SU-loaded nanoparticles within the H9c2 cell, we also performed 3D measurements of H9c2 cells along the z-axis. In the representative 3D (XYZ) scan, the same color scheme was applied as for the 2D (XY) scan above, and the 2D scan represents the second layer of the 3D scan (Figure 5). Thus, the mean spectra of two clusters containing SU-pSiNPs (red and orange spectra), the mean spectrum of the nucleus (blue), and the mean spectrum of the cytoplasm (green) were calculated, analyzed, compared, and plotted with their respective standard deviations (Figure 5B). Both red and orange spectra show a band at 518 cm^−1^, which is characteristic for pSiNPs, although the intensities are significantly weaker with respect to the remaining signals of the cytoplasmic components, especially compared to the reference spectra of nc-Si. Besides, the Si-characteristic Raman band has undergone a slight Raman shift from 520 to 518 cm^−1^ (compared to the reference spectra of Si in Figure 3). Two low-intensity Raman bands at 1320 cm^−1^ and 1569 cm^−1^ refer to SU-loaded pSiNPs. The changes in drug-specific vibrational fingerprints and the decrease in Raman intensities can be explained by the interaction of the nanocontainers and the drug with cellular components, pSiNP dissolution, and subsequent drug release (Tolstik et al., 2016b; Maximchik et al., 2019). The SU-specific peaks within the cytoplasm are of low intensity, making a pixel-wise identification challenging. Slight changes in laser intensity, the applied drug concentration, the focus plane, and other parameters can impact on the visibility/identification of their peaks. Nevertheless, our analyses validated the uptake of SU-pSiNPs, i.e., SU and pSiNPs, into the cells. Besides, the nuclear spectrum (in blue) does not contain any Raman band of Si, in contrast to the cytoplasm, even though the cellular Raman spectrum of Si has a slightly decreased Raman peak intensity around 518 cm^−1^ that is obviously due to a low pSiNP contribution. Of note, the Raman signal was integrated with 500 nm per pixel in spatial dimensions, which significantly extends the size of pSiNPs. Consequently, the silicon signal in the cytoplasmic spectrum can be a result of a small amount of randomly distributed pSiNPs or an agglomeration of pSiNPs. The high contribution of the orange cluster in the top layers, close to the cell membrane, validates the uptake of SU-pSiNPs into the cytoplasm and was found with higher concentrations close to the cell membrane after 24 h of incubation. Thus, this 3D scan with spectral information of both pSiNPs and SU further proves that the SU-loaded NPs were taken up by the cells and can be well localized by the application of Raman spectroscopy, and in principle the cells can be re-used for further analyses, e.g., immunofluorescence or MALDI mass spectrometry imaging (Ryabchykov et al., 2018). The application of Raman imaging *in vivo* would be of great benefit for clinical applications (Caspers et al., 2003; Cordero et al., 2018). Thus, first fiber-optic-based prototypes for tissue characterization are under development. However, there are several technical hurdles that need further sophisticated technical developments to translate Raman spectroscopy into clinical use.

### 3.3 Toxicity studies of pSiNPs, SU, and SU-loaded pSiNPs in cancer and cardiac muscle cells

For the evaluation of pSiNPs and their impact on the toxicity of SU, we examined H9c2 cells along with human colon carcinoma cell line HCT 116 as SU “target” cells, which are commonly used in the gastrointestinal oncology research (Sun et al., 2012). To assess whether the SU-loaded nanoparticles are similarly efficient to reduce cell survival as pure SU, we analyzed different markers of cell death and studied the toxicity of unloaded nanoparticles (NP) on H9c2 cells. The cleavage of the effector caspase-3 and its substrate PARP [poly (ADPribose) polymerase] was evaluated by WB analyses. These proteins are well-known apoptotic markers: active caspase-3 is a key effector enzyme in apoptotic signaling, which cleaves several substrates including PARP. PARP is engaged in the repair of DNA damage, and its cleavage by caspase-3 reflects the intensity of apoptosis (Lazebnik et al., 1994; Crowley et al., 2016). Cells were incubated with SU-loaded nanoparticles (concentration range from 5 to 50 μM) or SU (in concentrations of 5 and 10 μM) for 24, 48, and 72 h. As was shown previously, the pSiNPs themselves did not show cell toxic properties up to a concentration of 700 μg/mL in HCT 116 cells (Maximchik et al., 2019), thus allowing us to variate the concentration of SU loaded into the nanoparticles for further cytotoxicity studies to higher content of nanoparticles.

**FIGURE 6:**
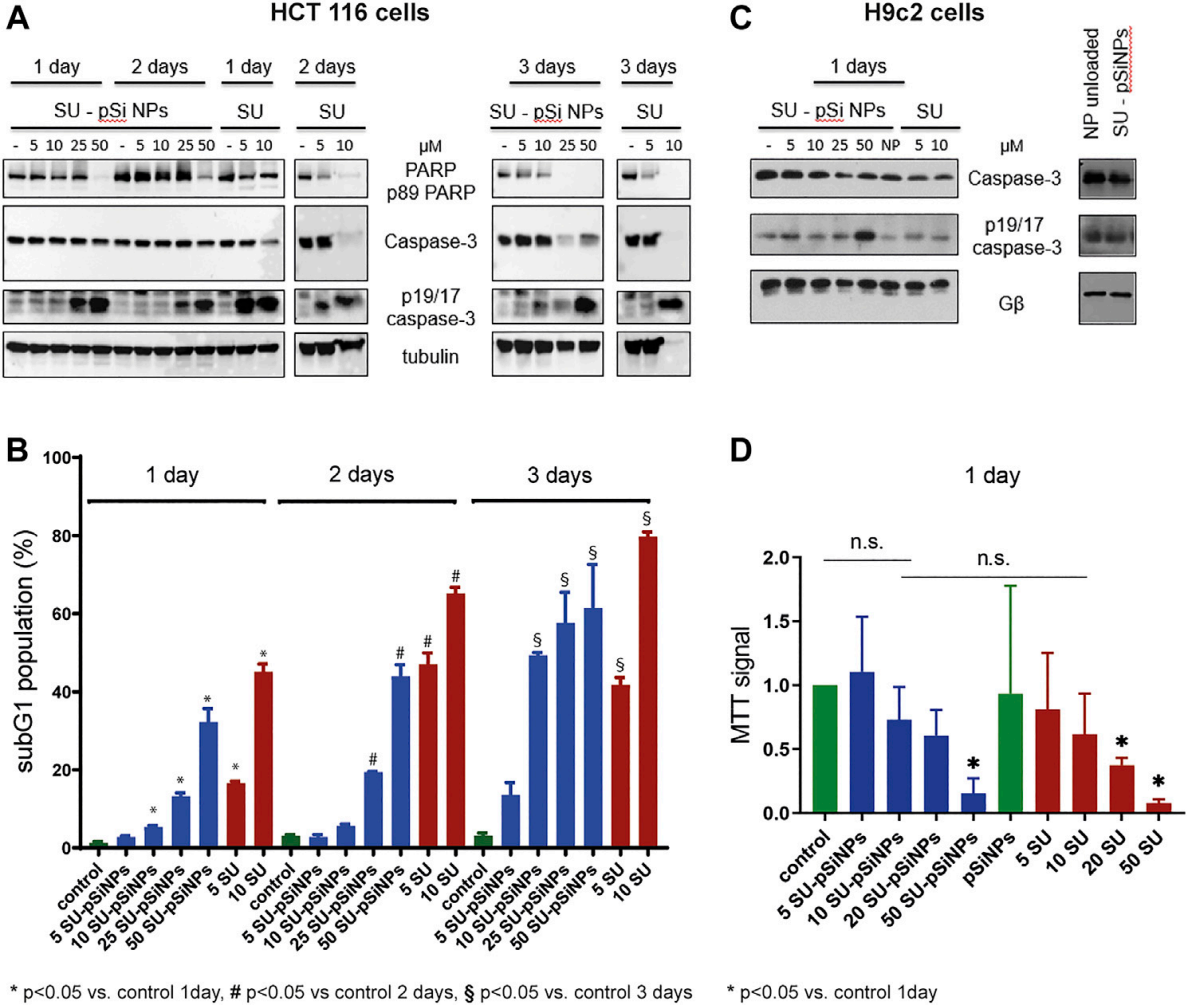
**(A–D)** HCT 116 cells and H9c2 cells were treated with sunitinib-loaded nanoparticles (SU-pSiNP) or pure sunitinib (SU) or unloaded nanoparticles (NP unloaded, **(C)**, right panel) in the indicated concentrations (μM) and for the indicated time intervals. In **(A)** and **(C),** the representative WB of expression levels of apoptotic marker proteins PARP, caspase-3, and cleaved caspase (p19/17 fragments of caspase-3, p19/17 caspase-3) are shown. Gβ and tubulin were used as loading controls. Blots were reproduced at least three times with similar results. **(B)** The histogram of flow cytometric analysis data for HCT 116 cells using subG1 assay; %: percent of SubG1 population. Results are shown as mean values ± standard deviation (SD), *n* = 3, *p < 0.05 vs. control 1 day, #*p* < 0.05 vs. control 2 days, §*p* < 0.05 vs. control 3 days, n.s. not significant. **(D)** MTT cell viability assay of H9c2 cells upon treatment with SU-pSiNPs and pure SU in indicated concentrations (μM) for 1 day. Results are presented as mean values ± standard deviation (SD), *n* = 3, *p < 0.05 vs. untreated control and pSiNPs-treated group. Ordinary one-way ANOVA was applied with Dunnett test to correct for multiple comparison.

According to the WB analyses, the treatment of HCT 116 with 5 or 10 μM of SU for 24 or 48 h caused an increase of cleaved caspase-3 (p19/p17 caspase-3). Also, slight decreases of procaspase-3 and full PARP were detected after 24 h and more pronounced decreases after 48 h of treatment. These data suggest the induction of apoptosis in HCT 116 cells by SU. In contrast, at least 25 μM of SU loaded in pSiNPs was needed to trigger a comparable increase of cleaved caspase-3 (p19/p17 caspase-3) (Figure 6A). After a 72 h treatment of HCT 116 cells with SU loaded in pSiNPs, 10 μM of SU appears to induce apoptotic effects as well, i.e., the signal for cleaved caspase-3 was increased and the signal for full PARP was decreased, which may suggest a delayed toxic effect of SU if loaded in pSiNPs.

To further validate these results, we performed flow cytometric analyses assessing the subG1 cell population, which monitors cells with accumulations of small DNA fragments in late stages of apoptosis due to increased endonuclease activity. The relative proportion of the subG1 population reflects the percentage of apoptotic cells (Plesca et al., 2008). Cell death analyses using subG1 test have revealed that the incubation of HCT 116 cells with SU (5 and 10 μM) for 24, 48, and 72 h significantly increased the subG1 population compared to the untreated controls (Figure 6B). Similarly as seen for the apoptotic markers in the WB analyses of HCT 116 cells, a delayed apoptotic response was induced by SU loaded in pSiNPs compared to SU treatment without the use of nanocontainers (see Figure 6B). While the cells treated with SU alone reached the maximum of apoptotic cells after 48 h, SU loaded in nanocontainers showed a further increase of apoptotic cells after 72 h (compare, e.g., the 10 μM condition after 48 and 72 h for SU alone and SU-pSiNPs).

Further, the toxicity of the chemotherapeutic agent SU was studied *in vitro* using the cardiomyoblast cell line H9c2 as a model system for cardiomyocyte toxicity (Merches et al., 2022). WB of cleaved caspase-3 (p19/p17 caspase-3) and caspase-3 revealed slight changes after a 24 h treatment of SU loaded in pSiNPs or SU alone (Figure 6C **);** the effects were more pronounced when the cells were treated with higher concentration of SU-pSiNPs (50 μM), similar to that seen in HCT 116 cells. In line with these results, the MTT test revealed a significant decrease in the viability of H9c2 cells, only after a 24 h treatment with 50 μM of SU-pSiNPs or 20 or 50 μM of SU alone (Figure 6D).

Taken together, our results show a time shift in the induction of a comparable level of apoptosis in HCT 116 cells by pure SU versus SU loaded in nanoparticles, which is most likely due to the sustained release of the drug from the nanoparticles that slows down cellular distribution of the drug. H9c2 cells seemed to be relatively resistant to SU treatment since at least 20 μM of pure SU was needed to induce cell death in H9c2 cells after 24 h treatment. Furthermore, the application of SU via pSiNPs or purely SU hardly affected the outcome on the cell survival. Even though, the cardiotoxicity of SU was hardly reduced by pSiNPs, WB analyses showed that pure pSiNPs are very well tolerated by these cardiomyoblast cells at least up to a concentration of 200 μg/ml as shown in Figure 6C, right panel. No accumulation of cleaved caspase-3 (p19/p17 caspase-3) or cleavage of caspase-3 was detectable after 24 h of incubation with nanoparticles. Thus, pSiNPs loaded with SU might be considered as a promising approach as drug depot with sustained drug release.

As cardiotoxicity of SU is linked to changes at mitochondrial level (Bouitbir et al., 2019), we aimed to study the impact of SU in comparison to SU-pSiNPs on these sensitive organelles. We thus analyzed the mitochondrial distribution as an early indicator for apoptotic changes (Karbowski and Youle, 2003). Therefore, confocal fluorescence microscopy was applied for H9c2 cells to study SU’s impact on the mitochondrial network. The cells were stained with MitoTracker^®^ Orange (λ_ex_ = 552 nm) and incubated with SU or SU-loaded nanoparticles in concentrations between 5 and 50 μM for 24 h (Supplementary Figure S1). The images were analyzed as described in Haupt et al. (2022). These analyses suggest that the mitochondrial network might be affected by SU. Cells treated with SU or SU-pSiNPs at concentrations shown to be toxic (20 and 50 μM; Figures 6C,D) were analyzed and they indeed revealed a reduction of the proportion of mitochondria within “networks” if pure SU was applied (depicted in red) compared to the incubation with SU loaded in nanocontainers (depicted in blue). These data indeed suggest that there may be a slight prevention of cell death if nanocontainers are used for SU administration; however, these data cannot exclude that the SU-induced cardiotoxicity is just delayed by nanoparticles since H9c2 cells were not yet investigated for longer than 24 h. Representative images are shown in Supplementary Figure S1B, where the mitochondrial network seems to have a rather comparable structure for control and SU-treated cells in concentrations up to 10 μM applied alone or in nanocontainers. The treatment with pure SU at higher concentrations (20 and 50 μM) caused a negative impact on the mitochondrial structures that were approved by quantitative analysis in Supplementary Figure S1A. A similar effect is visible for SU-pSiNPs treatment for 50 μM (but not 20 μM) of SU, validating therewith the quantitative analysis in the Supplementary Figure S1A.

Summing up the results, flow cytofluorometry and WB analysis showed that SU induces death of cancer and cardiac cells. Our experiments hint to apoptosis as a mode of programmed cell death and are in line with results that show SU-induced apoptosis in different cell types (Lin et al., 2011; Guo et al., 2021). However, further studies would be needed to rigorously assess the mode and time course of cytotoxicity. This cytotoxic effect in cancer cells was significantly delayed at comparable concentrations of the drug delivered in nanocontainers. Thus far, the experiments suggest that SU delivery via nanocontainers did not prevent cardiomyocyte death. The pSiNPs do not seem to be suitable for the prevention of cardiotoxicity per se; however, future studies will show whether functionalization of the nanocontainers for targeted drug delivery to cancer cells may reduce cardiotoxicity or whether intracellular drug depots may improve the effects of drugs, e.g., applied for heart failure. Thus, along with the assessed *in vitro* cardiosafety of pSiNPs in this study, new perspectives for their application, e.g., for controlled drug delivery with slow and sustained release, will open new prospects for patients with chronic heart diseases.

## 4 Conclusion

In this article, we monitored the uptake and distribution of the anticancer drug sunitinib (SU), delivered in nanocontainers based on pSiNPs or as a dissolved pure compound, with a set of biophotonics and analytical techniques. The first results suggest that pSiNPs are effectively taken up into the cytoplasm of several cell types and can build an intracellular drug depot that facilitates continuous drug release. Porous silicon nanoparticles have great advantages for the delivery of the drug. Since they exhibit luminescent properties and a characteristic Raman band that does not overlap with the Raman bands of cellular organelles, these nanoparticles can easily be detected in cells and within the cellular compartment by Raman micro-spectroscopy. 2D and 3D Raman micro-spectroscopy imaging has the advantage of enabling the label-free visualization of drug depots and potentially the drug release as well.

Flow cytofluorometry and WB analysis further showed that sunitinib triggers apoptotic death of cancer and cardiac cells. This cytotoxic effect in cancer cells was significantly delayed if SU was delivered in nanocontainers. The observed slight differences of cell survival and the integrity of mitochondrial networks whether SU was applied to H9c2 cells within nanoparticles or not may also be due to a delayed toxicity. Even if pSiNPs may not be suitable for the prevention of cardiotoxicity *per se*, future studies will show whether functionalization of the nanocontainers for targeted drug delivery to cancer cells may reduce cardiotoxicity or whether intracellular drug depots may improve the effects of drugs, e.g., applied for heart failure. Thus, along with the here assessed *in vitro* cardiosafety of pSiNPs, new perspectives for their application, e.g., for controlled drug delivery with slow and sustained release, will open new prospects for pharmacological treatment options.

## Data Availability

The raw data supporting the conclusions of this article will be made available by the authors, without undue reservation.

## Supplementary material

Supplementary Figure S1 Analysis and representative images of H9c2 cells treated for 24 h with pure sunitinib (SU) or with sunitinib loaded in nanoparticles (SU-pSiNPs) with the indicated concentrations. The H9c2 cells were stained with MitoTracker™ Orange to visualize mitochondrial network complexity. **(A)** Percentage of mitochondrial networks at different concentrations and the ways of treatment: SU with pSiNPs depicted in blue and without pSiNPs depicted in red. Results are presented as mean ± standard deviation (SD) and shown as blocks for each case. For the quantitative analysis of the mitochondrial structures, Fiji program was used to preprocess the measurements, to create a segmentation map of the mitochondria and to perform a binary skeleton analysis, following the protocol presented by Haupt et al., 2022. Based on this, the percentage of branched mitochondrial networks was calculated and compared through the different concentrations. (B) Representative fluorescence images of H9c2 stained with MitoTracker™ Orange to visualize mitochondrial network; the scale bar was 20 μm. The H9c2 cells were treated for 24 h with pure sunitinib (SU, *upper row*) or with sunitinib loaded in nanoparticles (SU-pSiNPs, *lower row*). At least 5 to 10 cells of each SU concentration and each pSiNPs were analyzed and the representative images are shown.
