# Supplementary figures and images for: Raman and fluorescence micro-spectroscopy applied for the monitoring of sunitinib-loaded porous silicon nanocontainers in cardiac cells
Source: Front Pharmacol. 2022 Aug 9;13:962763. doi: 10.3389/fphar.2022.962763 (PMC9397571; doi:10.3389/fphar.2022.962763)

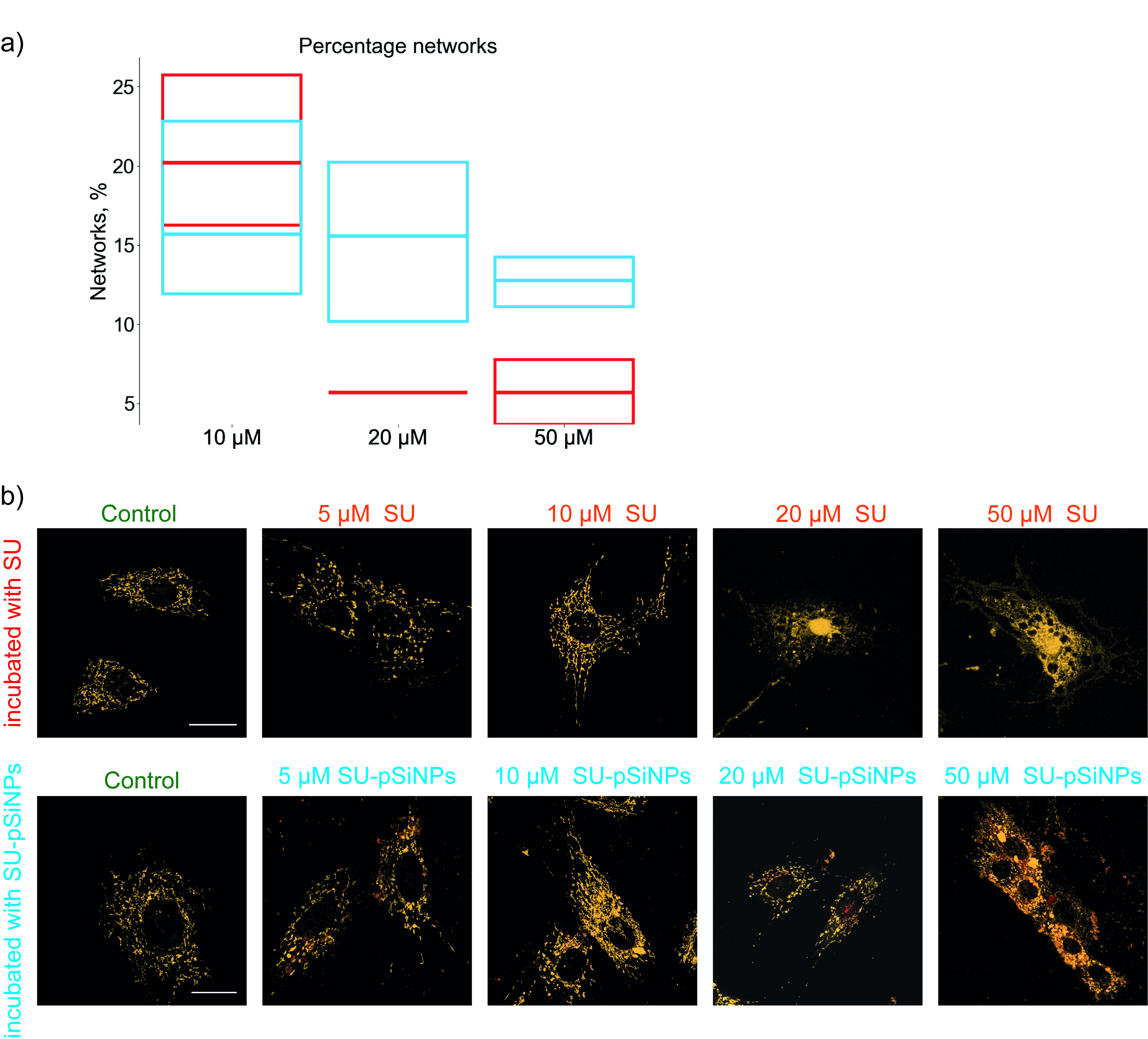

Supplement: Supplementary file 1 [file Image1.tif]
